# Predictors of triple therapy treatment failure among *H. pylori* infected patients attending at a tertiary hospital in Northwest Tanzania: a prospective study

**DOI:** 10.1186/s12879-019-4085-1

**Published:** 2019-05-21

**Authors:** Hyasinta Jaka, Andreas Mueller, Christa Kasang, Stephen E. Mshana

**Affiliations:** 1 Department of Internal medicine-Gastroenterology and Hepatology unit, Catholic University of Health and Allied Sciences, P.O.Box 1464, Bugando, Mwanza, Tanzania; 2 Tropenmedizin, Missionsärztliche Klinik, Salvatorstr. 7, 97074 Würzburg, Germany; 3 Medical Mission Institute, Hermann Schell Str. 7, 97074 Würzburg, Germany; 4 Department of Microbiology and Immunology, Catholic University of Health and Allied Sciences, P.O.Box 1464, Bugando, Mwanza, Tanzania

**Keywords:** *Helicobacter pylori*, Eradication, Stool antigen, Clarithromycin, Adherence, Tanzania

## Abstract

**Background:**

*Helicobacter pylori (H.pylori)* infection is a common medical problem in resource limited areas. The treatment outcome after triple therapy has not been well studied in developing countries and preliminary data suggests a high rate of treatment failure. This study investigated the triple therapy treatment failure rate and associated factors among dyspeptic patients receiving *H. pylori* first line therapy at a tertiary hospital, Tanzania.

**Methods:**

A prospective study in the Gastroenterology unit of the Bugando Medical Centre (BMC) was conducted between October 2015 and May 2017. All dyspeptic patients with stool antigen tests positive for *H.pylori* were given first line therapy, and stool antigen testing was repeated within 7 days and 5 weeks after completion of the treatment*.* Biopsies were taken before initiation of therapy and analysed for clarithromycin and quinolone resistance mutations using polymerise chain reaction (PCR) and sequencing. Adherence and other social-demographic characteristics were documented.

**Results:**

A total of 210 patients were enrolled; the median age was 35 years (interquartile range, 27–48). First line treatment failure as defined by positive stool antigen 5 weeks post treatment was observed in 65/210 (31%) of patients. Independent predictors of first line treatment failure were presence of clarithromycin resistance mutations (OR: 23.12, 95% CI (9.38–56.98), *P* < 0.001) and poor adherence (OR: 7.39, 95% CI (3.25–16.77), *P* < 0.001). The sensitivity and specificity of stool antigen testing within 7 days after completion therapy in detecting treatment failure was 100 and 93.2%, respectively.

**Conclusion:**

Nearly one-third of patients with clarithromycin resistance mutations and poor adherence develop first line treatment failure. Routine stool antigen testing within seven days after completion of therapy can be considered in order to initiate second line treatment early to prevent associated morbidities.

## Background

*Helicobacter pylori* is one of the most prevalent organism that can lead to erosions, ulcerations and cancers [1]. Overall the prevalence of *H.pylori* infection is about 44.3% worldwide and is more in developing countries than in developed countries [2]. The International Agency for Research on Cancer (IARC), which is part of the World Health Organization (WHO) declared *H pylori* as class I carcinogen [3].

*H.pylori* infected persons have a 10 to 20% lifetime risk of developing ulcer and a 1 to 2% lifetime risk of developing gastric cancer [4]. With increase resistance to clarithromycin worldwide there is an increased risk of first line *H. pylori* treatment failure [5, 6]. Treatment guidelines for the management of *H. pylori* infection differ among countries and depend on local susceptibility patterns [7]. The efficacy of standard 7 days therapy is decreasing globally [8, 9]. Eradication rates of *H.pylori* have been found to be less than 80% in some countries [10–12]. The *H. pylori* treatment failure has been linked to infections with antibiotic resistant strains [13–16], host genetic polymorphism in the cytochrome that may affect proton pump inhibitor pharmacokinetics (CYP2C19), poor adherence, short duration of therapy and smoking [17–19].

Previous efforts to improve the eradication of *H. pylori* have focused on increasing the numbers and types of drugs in the regimens and prolongation of the duration of standard triple therapy, none of which achieved higher eradication rates [20–22]. Other newer efforts include the implementation of concomitant (Proton pump inhibitor, amoxicillin, clarithromycin, and a nitroimidazole (tinidazole or metronidazole) given together for 3–10 days) and hybrid therapies (Proton pump inhibitor and amoxicillin for 7 days followed by another 7 days of Proton pump inhibitor, amoxicillin, clarithromycin, and a nitroimidazole) [23–26]. These therapies have resulted in eradication rates of 85 to 94%, and are currently advocated in places with greater than 15% clarithromycin resistance [27–30].

*H. pylori* treatment outcome and associated factors have not well been studied in East African countries. To-date there is the report of 10 patients who were investigated in 1999 from Ethiopia [12]. This study has provided the magnitude and factors associated with treatment failure in patients treated with first line regimen from developing country and has documented sensitivity and specificity of stool antigen testing within 7 days in detecting treatment failure. These information are very crucial in devising appropriate strategies to improve the outcome of *H.pylori* treatment.

## Methods

### Study population

This prospective study was done in the Gastroenterology and Hepatology unit of the Bugando Medical Centre a tertiary hospital located in the North-western Tanzania between October 2015 and May 2017. All dyspeptic patients who were planned to undergo esophagogastroduodenoscopy (EGD) aged 18 yrs. and above were invited in the study. Dyspepsia was defined using ROME criteria [31]. The study enrolled all patients who were *H. pylori* stool antigen positive with no history of antibiotic use in the past 30 days. The primary outcome was treatment failure which was defined as positive *H.pylori* stool antigen test 5 weeks after completion of the treatment [32].

### Specimen collection and transportation

Participants who fulfilled inclusion criteria were serially enrolled. The stool antigen tests for *H.pylori* was performed using HpSA antigen test (SD- Bioline, *H.pylori* Ag Rapid test, Germany). In addition, on the same day the EGD was performed, two biopsies from antrum and fundus were taken. All patients who had positive stool antigen results were given a prescription for triple therapy according to standard treatment guidelines. A low cost regimen for limited-resource settings was prescribed to all patients (proton pump inhibitor, clarithromycin 500 mg and metronidazole500mg /Amoxicillin 1 g twice a day for 10 days), for those who failed the first line were given second line which included proton pump inhibitor, levofloxacin and amoxicillin [33]. Biopsies were taken followed by DNA extraction as described previously [34]. Amplification and sequencing of the clarithromycin resistance-determining regions to detect clarithromycin resistance mutations was done at the Department of Medical Microbiology, University of Gottingen, Germany.

### Molecular testing

QIAamp DNA mini tissue extraction (Qiagen SA, Courtaboeuf, France) was used to extract DNA following the manufacturer instructions. *H. pylori* clarithromycin mutations were determined by amplification (Light Cycler- Roche) and sequencing of a 267 base pair (bp) fragment of the *H. pylori* 23S rRNA using oligonucleotides HPY-A: 5-CGCATGATATTCCCATTAGCAGT and HPY-S: 5-AGGTTAAGAGGATGCGTCAGTC [35]. The PCR was carried out in 40 cycles with denaturation process at 95 °C for 10 s, annealing at 60 °C for 10 s and extension at 72 °C for 20 s. In each cycle melting curve analysis was performed. Detection of mutations within the 23S gene was done by DNA sequencing followed with alignment with the wild type allele using the Geneious software package (Version 8.0.4 available from www.geneious.com (Biomatters, Ltd). DNA sequences were analysed for point mutations that are known to confer clarithromycin resistance at positions G2141, A2142, A2143 and A2144.

### Follow up of patients

All enrolled patients had follow-up stool testing done twice: immediately (within seven days) after completion of medications and 5 weeks after completion of the treatment. Those with positive stool antigen 5 weeks after completion of treatment were declared as treatment failure. At the follow-up visits, the adherence was assessed by independent researcher as described by Gahi et al. [36]. In this scale the following questions were asked; in the past ten days, how often did you take your medications as the doctor prescribed?" Probable responses were; “All of the time” (100%), “Nearly all of the time” (90%), “Most of the time” (75%), “About half the time” (50%), or “Less than half the time” (< 50%). Poor adherence was defined as taking medications as prescribed 75% of the time or less.

All patients with treatment failure [30] were given a second-line treatment regimen according to the standard guidelines, tailored by resistance mutations identified.

### Data analysis

Data were entered in the computer and analyzed using STATA version 13. The main outcome in this study was treatment failure. The categorical variables such as sex, were summarized as proportions while continuous variables such age were summarized as median with interquartile range (IQR). The univariate analysis was done which described the relationship between individual factors and treatment failure. Furthermore, multivariate analysis was done to eliminate confounders, odds ratios (OR) with respective 95% confidence intervals (CI) were computed. In all analyses, factors with a *P* < 0.05 were considered statistically significant. Furthermore, sensitivity and specificity of immediate testing of stool antigen using determination after five weeks post treatment as gold standard was done**.**

## Results

**Fig. 1:**
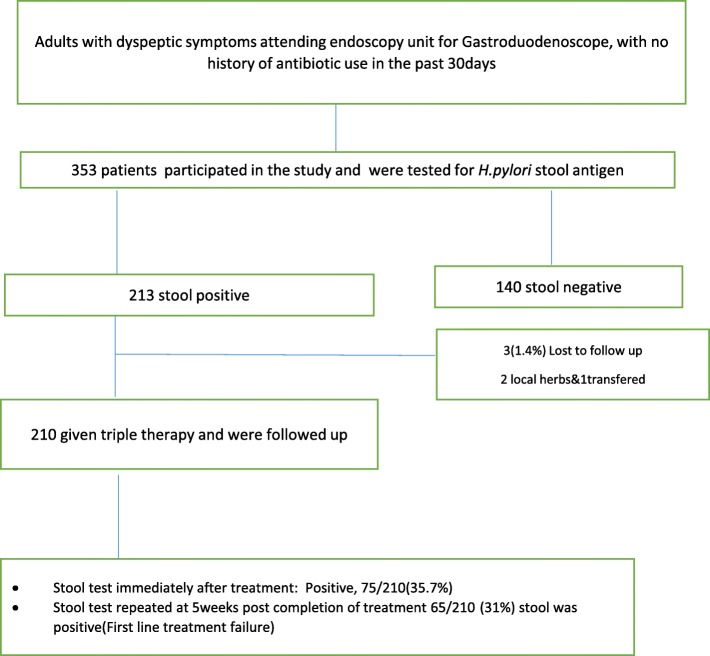
Flow chart of patient recruitment and follow up

Total of 353 participants were invited and participated in the study. A total of 213 dyspeptic patients who tested positive for *H.pylori* stool antigen were included in the study. Out of 213 patients who were *H. pylori* stool antigen positive (Fig. 1), 3/213(1.4%) were lost to follow up (one was transferred to another region, two patients decided to use local herbs). A total of 210 participants were followed to completion, their median age was 35 yrs. (IQR 27–48). The endoscopy findings of 210 participants indicated that the majority had gastritis/duodenitis (Table 1).

**Table 1:** Endoscopic findings among 213 patients included in the study at baseline

Endoscopic findings	Number of patients (%)
Gastritis/Duodenitis	147(69.0%)
Gastric/Duodenal ulcer	53(24.8%)
Normal	9(4.22%)
Gastric outlet obstruction	4(1.98%)
Total	213(100%)

### *H.pylori* results on follow up

Immediately after completion of treatment (within seven days), all patients were tested for *H.pylori* infection, 75/210 (35.7%) were *H. pylori* stool antigen positive. Five weeks after completion of treatment, 65/210 (30.9, 95% CI; 24.6–37.2) were still stool antigen positive and were considered as treatment failure.

The sensitivity of immediately testing (within 7 days of completion treatment) in detecting treatment failure was 100% with the specificity of 93.2%, positive predictive value of 86.7% and negative predictive value of 100%. Sixty five (30.9%) patients who had treatment failure were treated with a second line or concomitant regimen [30] based on their mutation results. Among these 29/65(44.6%) failed the treatment and were given sorted drugs which achieved eradication. A total of 42/210(19.9%) reported side effects; some had more than one side effects. Side effects observed were abdominal discomfort 32/210(15.2%), nausea 6/210(2.8%), vomiting 2/210(0.9%) and dizziness 2/210(0.9%).

### Factors associated with treatment failure

Among 210 patients, 152/210(72.3%) were tested for the clarithromycin resistance mutations (Table 2). Treatment failure was observed in 46/152(30.3%) patients with clarithromycin mutations while only 16(15.1%) patients in the group with no mutations had treatment failure (*p* < 0.001). Common mutations detected were A2143G 26/46(56.5%) and A2142G.

**Table 2:** Factors associated with triple therapy failure among 210 patients attended at the Bugando Medical Centre, Mwanza

Characteristics	Total number 210	First line treatment failure	Univariate		Multivariate	
			OR(95% CI)	*P*-value	OR(95% CI)	*P*-value
Age in years	35(27–48)		1(0.96–1.03)	0.983		
Gender						
Male	99	32(49.2%)	1			
Female	111	33(50.8%)	0.810 (0.451–1.455)	0.481		
Adherence						
Good	177	43(23.73%)	1		1	
poor	33	22(69.70%)	7(3.258–16.772)	< 0.001	27(7.25–108)	< 0.001
Clarithromycin Mutations						
Not tested	58					
No mutations	106	16(15.09%)	1		1	
Mutations	46	37(80.43%)	23(9.38–56.98)	< 0.001	69(19.6–249.6)	< 0.001

On the logistic regression analysis(Table 2), patients with isolates harbouring clarithromycin mutations were more likely to be *H. pylori* stool antigen positive 5 weeks post treatment (OR: 69.96, 95% CI; (19.61–249), *P* < 0.001) than those with no mutations. In addition, patients with poor adherence (OR: 7, 95% CI: 3.258–16.772, *P* < 0.001) were also more likely to be *H.pylori* stool antigen positive 5 weeks post treatment than those with good adherence. By multivariate logistic regression analysis, poor adherence (*P* < 0.001) and presence of clarithromycin resistance mutations (*P* < 0.001) significantly predicted treatment failure (Table 2).

## Discussion

Standard *H.pylori* triple therapy has been a regimen of choice for many years. Unfortunately there is a decline in its efficacy hence clinicians have replaced standard triple therapy with sequential, concomitant and hybrid therapy (modified sequential) to increase cure rate [26, 37, 38]. It should be noted that the efficacy of standard triple therapies, which is still recommended in most guidelines, has decreased to < 80% in different countries [39–44].

As a general standard for the treatment of *H.pylori*, the anti- *H.pylori* therapeutic regimens should have an eradication rate of ≥90% [45]. Here, we report an alarming treatment failure rate of over 30% among patients with *H. pylori* infection seeking medical care in a tertiary hospital, Tanzania. This study for the first time in Tanzania has documented the *H. pylori* first line treatment failure among patients who were not previously on antibiotic. The treatment failure reported in the current study is significantly lower than what was reported in Egypt, this could be explained by the choice of patients. In current study, dyspeptic patients included were not on antibiotics within 30 days before the stool test was done while in Egyptian study the patients included were those who presented with dyspepsia without considering the prior use of antibiotics [46]. It should be noted that secondary clarithromycin resistance has been found to be significantly higher than primary resistance [47]. When compared to a study in Rwanda which reported treatment failure of 20% [44], no specific reason was elucidated to explain this variations pointing to geographical differences in the magnitude of resistance. High resistance to clarithromycin draws the urgent need to change treatment strategies in order to improve the treatment outcome in our setting. In addition, more studies are needed to explore drivers of the resistance.

The efficacy of the first line treatment therapy for *H. pylori* is decreasing in different parts of the world [48, 49]. As observed in the current study, clarithromycin resistance has been implicated as the potential factor for the first line treatment failure in many studies [39, 42, 50–52]. A study from Rwanda [44] also observed that clarithromycin resistance was a significant factor that predicted treatment failure. It should be noted that clarithromycin mutations often predict treatment failure of clarithromycin-based regimens [53, 54]. This was the case in the current study, with big odd ratio and 95% CI being reported due to the fact that there was significant more participants in the group with no mutation than that with mutation.

Good adherence has been found to predict good outcome of medication [42, 55–57], this was also observed in the current study. Poor adherence always leads to the sub-therapeutic levels or sub-optimal dose resulting in poor clinical outcome [58]. Interestingly, a study looking at the role of adherence to medications found that close follow-up of patients did improve compliance and satisfaction for the patients but did not increase the *H. pylori* eradication rate [36, 59]. This might have been due to other factors like drug resistance which can affect the eradication of the infection. Despite age and sex being documented as factors associated with *H. pylori* treatment outcome, this was not the case in the present study [39] [60].

Standard practices for the management of *H. pylori* infection recommends that post treatment non-invasive tests to confirm the eradication of *H. pylori* must be performed 4 weeks or more after eradication therapy is completed. In current study, stool antigen test done within 7 days after treatment well predicted eradication and treatment failure (positive predictive value, 86.7%, with negative predictive value of 100%). This finding was also observed in the studies done in United States and Europe, whereby positive result on the stool antigen test 7 days after completion of therapy identified patients in whom eradication of *H. pylori* was unsuccessful [61]. These data suggest that earlier stool antigen follow-up testing would identify those who will fail therapy and allow early initiation of second-line treatment [62, 63].

## Conclusion

About one third of patients with clarithromycin resistance mutations and poor adherence developed first line treatment failure. Stool antigen testing within seven days after completion of therapy can be considered in order to initiate second treatment early to prevent associated morbidities. Strengthening adherence counselling before and during treatment is crucial in ensuring treatment success. More effort is needed in developing countries to ensure individual tailored treatment basing on the susceptibility patterns is practised.

## Abbreviations

BMC: Bugando Medical Centre
Bp: Base pair
CI: Confidence Interval
CUHAS: Catholic University of Health and Allied Health
DNA: Deoxyribonucleic acid
EGD: Esophagogastroduodenoscopy
IQR: Interquartile range
OR: Odds ratio
PCR: Polymerise chain reaction
